# Efficacy of peracetic acid and chlorine in managing *Salmonella* biofilms in irrigation loop systems

**DOI:** 10.1128/aem.01974-25

**Published:** 2025-12-19

**Authors:** Rawane Raad, Blanca Ruiz-Llacsahuanga, Charles Bency Appolon, Halle Greenbaum, Ruben Vinueza, Faith Critzer

**Affiliations:** 1 Department of Food Science and Technology, University of Georgia 1355, Athens, Georgia, USA; Universita degli Studi di Napoli Federico II, Portici, Italy

**Keywords:** irrigation water, distribution lines, *Salmonella*, fertilizer, sanitizer, crop production

## Abstract

Biofouling presents significant challenges to the crop production industry, notably reducing irrigation efficiency and potentially dispersing pathogens to irrigated crops. This study evaluated the efficacy of peracetic acid (PAA) and chlorine (Cl) against *Salmonella* biofilms in irrigation lines with or without fertilizers. Pond water (PW) with 2-4-1 fish emulsion (O), PW with 4-0-8 synthetic liquid fertilizer (S), or PW with no fertilizer (NoFert) was inoculated with 2 log CFU/mL of a rifampicin-resistant *Salmonella* cocktail. Inoculated water was then circulated through polyethylene loop irrigation system for a month. *Salmonella* populations both in the water and attached to the tubing were determined. Data showed that a single point of contamination from the water resulted in a biofilm formation with O and NoFert, but not the S treatments, after 3 days. Both PAA and Cl effectively reduced *Salmonella* populations for all fertilizer treatments in water samples. However, when no sanitizer was introduced to the line, bacterial dispersion resulted in the contamination of a subsequent irrigation event for the O treatments but not the S and NoFert treatments, which presented no microbial proliferation. Our findings suggest that O treatments resulted in persistent biofilm formation that could lead to contamination of irrigation water when no sanitizers are introduced. These studies provide insight into the behavior of foodborne pathogens in irrigation distribution systems.

**IMPORTANCE:**

The accumulation of bacteria in water distribution systems due to biofouling can lead to contamination, making it crucial to evaluate and implement effective mitigation measures to prevent these issues and ensure safe and efficient irrigation practices. The use of the 2-4-1 fish emulsion in-line may support the establishment of *Salmonella* biofilms and subsequent cross-contamination of irrigation water if not fully flushed from the system. This study demonstrates that PAA and Cl effectively reduce *Salmonella* contamination in water but will not eliminate populations in-line once biofilms are established.

## INTRODUCTION

Current data indicate that produce accounted for 50% of illnesses in the U.S. with *Salmonella enterica* being responsible for a majority of those outbreaks (1). The consumption of raw produce has been generally perceived as safe compared with other foods primarily of animal origin. However, vegetable production systems inherently present multiple points of vulnerability to foodborne pathogen contamination. This includes production and pre-harvest practices such as using contaminated irrigation water (2, 3). Irrigation water sources include groundwater and surface water. Groundwater resides in aquifers beneath the earth’s surface, whereas surface water includes ponds, lakes, or rivers. Surface water is commonly used in irrigation systems due to its accessibility and abundance in the southern U.S. However, it can easily be contaminated with endemic bacteria from the environment, potentially transferring pathogens to the soil or crop (2, 4–6). Fertilizers are often added to irrigation systems through fertigation, a common practice among commercial growers (7). Synthetic fertilizers, primarily Nitrogen (N), Phosphorus (P), and Potassium (K), are widely used in conventional agriculture, with nutrient levels indicated by numbers (e.g., 4-0-8 fertilizer contains 4% N, 0% P, and 8% K) (8). In contrast, organic farms adhere to the U.S. Department of Agriculture’s National Organic Program (NOP) guidelines, where biological amendments of animal origin, such as fish emulsion and blood meals, are used as fertilizers (9). Basic water distribution system components include valves, fittings, pumps, sprinklers, storage reservoirs, tanks, and the piping materials used in agricultural fields. This includes polyvinyl chloride (PVC), which is popular due to its durability and cost-effectiveness, and high-density polyethylene (HDPE), which is favored for its high strength and flexibility, making it suitable for diverse irrigation applications (10, 11). These materials are recognized for providing an ideal surface that promotes bacterial colonization and biofilm formation (12).

Biofilms are integrated multi-species cell populations that are embedded in a self-produced matrix of extracellular polymeric substances (EPS) (13). Multiple studies reported biofilm formation of *Salmonella* (14), *Pseudomonas* (15), and *Escherichia coli* (16) on HDPE surfaces. Gamri et al. (17) examined the impact of pipe materials on biofouling using synthetic wastewater. The study found that biofilm growth was more pronounced at velocities of 0.8 m/s and that although PVC pipes were less prone to bacterial accumulation compared with polyethylene pipes, they were still susceptible to biofilm formation (17). Additionally, *Salmonella* has been shown to persist in packing line materials such as stainless steel, PVC, and unfinished oak wood for an extended period of time (over 28 days) (18). The formation of biofilms in agricultural water distribution systems is unavoidable and can lead to several problems, including reduced irrigation efficiency due to pipeline blockages and compromised water safety (19).

Bacteria in the biofilm form are resistant to several stresses such as the use of sanitizers (20, 21). This includes chlorine (Cl) in the form of sodium or calcium hypochlorite and peracetic acid (PAA). These chemicals are often used for sanitation of equipment in food processing facilities (22, 23) or to treat agricultural water (24, 25). Several factors, including levels of organic matter formed, pH, water temperature, and concentration of the chemical used, can alter the effectiveness of a treatment against *Salmonella* biofilms (26). It is important for the produce industry to establish control measures to prevent biofilm formation in the irrigation pipelines throughout their production. PAA and Cl are both effective disinfectants used to reduce microbial contamination, but their effects are through different mechanisms (27–29). Cl is commonly used in drinking water distribution systems to resolve biofouling issues due to its availability and low cost (12). PAA, specifically in the form of SaniDate 12.0, is currently an EPA-registered product labeled for the reduction and control of Shiga toxin-producing *E. coli* (STEC), and *Salmonella enterica* in preharvest irrigation water (EPA Reg. No. 70299-18). Both disinfectants are influenced by factors such as concentration, contact time, and water characteristics, but PAA tends to dissipate quickly, whereas Cl’s effectiveness is impacted by water, pH, and temperature (28, 30).

Despite a few studies reporting the effectiveness of various disinfectant strategies in reducing clogging and bioaccumulation in piping systems (31, 32), there is a notable lack of research addressing foodborne pathogen biofilm formation in irrigation water distribution settings, particularly when synthetic fertilizers or fish emulsion are involved. Irrigation water has been the main focus as a source of contamination, but very little attention is paid to the sanitation of irrigation tubing to ensure that any organisms remaining in the line do not become a source of contamination. Therefore, the objective of this study was to evaluate the efficacy of sanitation control measures (Cl or PAA), which could be implemented by produce growers to manage the risk of *Salmonella* biofilms in an in-lab irrigation loop piping system with the presence of synthetic (S) or fish emulsion (O) fertilizers. This research focuses on increasing knowledge about the role of common sanitizers used for preharvest water in preventing the conditions that favor the biofilm formation of *Salmonella* in irrigation lines, leading to potential cross-contamination of the water. The insights gained could inform industry practices and guide future research efforts toward more holistic approaches in managing pathogen contamination in agricultural settings.

## RESULTS

### Injecting the line with fish emulsion resulted in *Salmonella* attachment and growth in drip tubes over time

**Fig 1:**
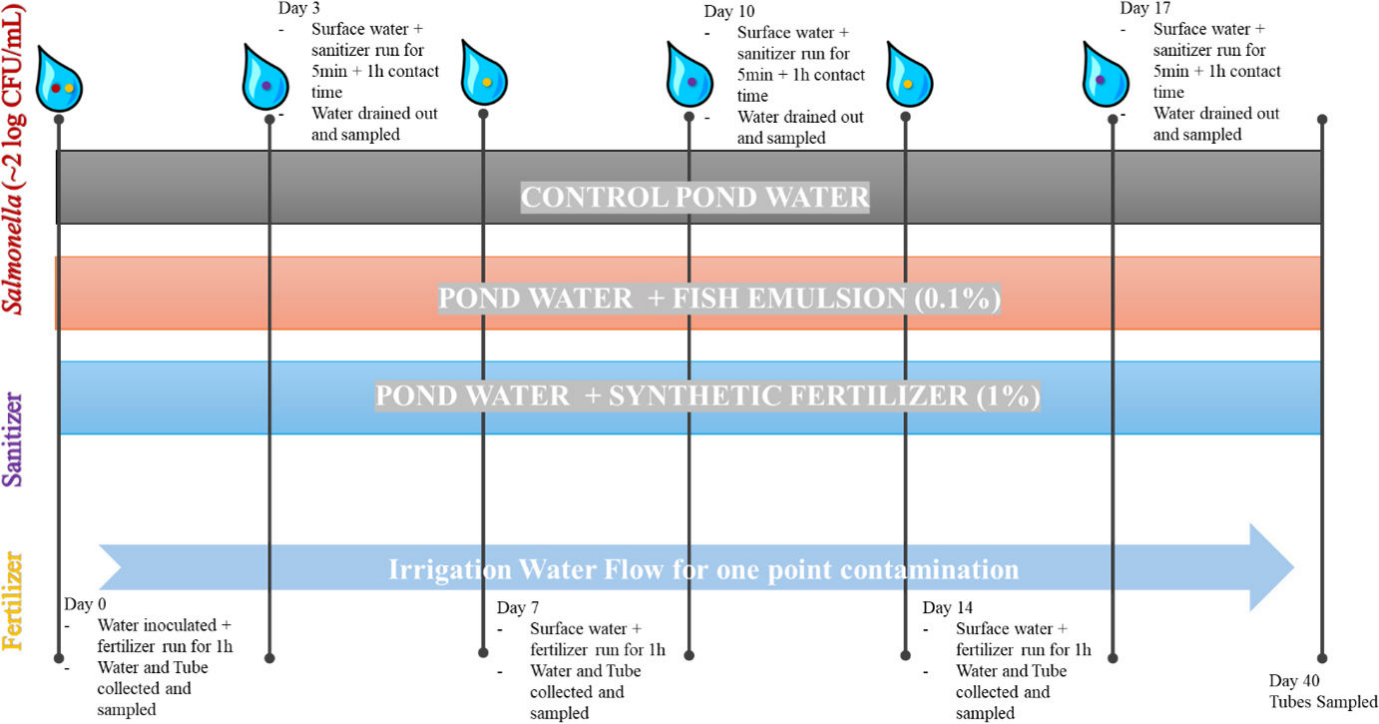
Timeline of experimental procedure for tubing circulated with either pond water (PW) injected with 1% (vol/vol) synthetic (4-0-8) fertilizer, PW injected with 0.1% (vol/vol) fish emulsion; or PW with no fertilizer (control pond water) during 40 days of sampling. Inoculated water was circulated on day 0 and then drained. On days 3, 10, and 17, PW was injected with sanitizers: peracetic acid or chlorine at 20 ppm each. On days 7 and 14, water was injected with its respective fertilizer. Tubes were kept until day 40 for sampling to mimic crop production month.

Across all treatment combinations, *Salmonella* populations in water remained consistent after 1 h of inoculated water circulation in the drip tubes on day 0 (*P* = 0.23; Table 1). It is important to note that sanitizers were introduced on day 3, not on day 0 (Fig. 1). On day 0, cross-contamination from the inoculated water to the drip tubes was evident in the 0.1% vol/vol fish emulsion treatments (O) regardless of sanitizer condition: No sanitizer: −0.31 log colony-forming unit (CFU)/tube, PAA: −0.08 log CFU/tube, and Cl: 0.18 log CFU/tube. Similarly, cross-contamination was evident for the synthetic (1% vol/vol) 4-0-8 (S) fertilizer treatment with no sanitizer: −0.68 log CFU/tube, PAA: −0.57, and Cl: −0.68 log CFU/tube. Although levels for the no fertilizer (NoFert) treatment were −0.26 CFU/tube for the no sanitizer, −0.51 log CFU/tube for the PAA, and −0.55 log CFU/tube for the Cl treatment combinations (Table 2). Regardless of the treatment, *Salmonella* populations attached to the drip tubes on day 0 were significantly different between the O and the NoFert (*P* = 0.01) and the O and the S (*P* ≤ 0.001) but not between the S and the NoFert treatments (*P* = 0.07).

**TABLE 1:** *Salmonella* population (log CFU/mL) mean ± standard deviation in pond water inoculated from time 0 to 1 h, at different fertilizer conditions: 0.1% vol/vol fish 2-4-1 emulsion (O), 1% vol/vol synthetic liquid 4-0-8 fertilizer (S), or pond water only (NoFert)^*a*^

Fertilizer	Sanitizer	Time (h)	
		0	1
O	PAA	2.33 ± 0.10	2.31 ± 0.04
	Cl	2.35 ± 0.15	2.34 ± 0.05
	No sanitizer	2.33 ± 0.15	2.32 ± 0.08
S	PAA	2.25 ± 0.13	2.13 ± 0.17
	Cl	1.81 ± 0.69	2.09 ± 0.32
	No sanitizer	1.89 ± 0.45	2.06 ± 0.22
NoFert	PAA	2.34 ± 0.09	2.12 ± 0.73
	Cl	2.35 ± 0.12	2.34 ± 0.10
	No sanitizer	2.31 ± 0.18	2.33 ± 0.12

^*a*^ Each separated based on their sanitizer condition: peracetic acid (PAA) or chlorine (Cl) at 20 ppm each or no sanitizer, which served as control. Limit of detection: −1 log CFU/mL. *N *= 9 per treatment combination.

**TABLE 2:** *Salmonella* population (log CFU/tube) mean ± standard deviation in tubing samples over time at different fertilizer conditions: 0.1% vol/vol fish 2-4-1 emulsion (O), 1% vol/vol synthetic liquid 4-0-8 fertilizer (S), or pond water only (NoFert)^*a*^

Fertilizer	Sanitizer	Day 0	Day 7	Day 14	Day 40
O	No sanitizer	−0.31 ± 0.45 Aa*	5.45 ± 0.82 Ba	5.49 ± 1.00 Ba	4.29 ± 1.52 Ba
	PAA	−0.08 ± 0.43 Aa*	1.49 ± 1.09 Bb	1.51 ± 1.35 Bb	0.40 ± 1.22 ABb
	Cl	0.18 ± 0.53 Aa*	0.43 ± 0.65 Ab	0.09 ± 0.68 Ab	−0.75 ± 0.15 Bb
S	No sanitizer	−0.68 ± 0.27 Aa**	−0.82 ± 0.00 Ba	−0.82 ± 0.00 Ba	−0.82 ± 0.00 Ba
	PAA	−0.57 ± 0.23 Aa**	−0.82 ± 0.00 Ba	−0.82 ± 0.00 Ba	−0.82 ± 0.00 Ba
	Cl	−0.68 ± 0.31 A**	−0.82 ± 0.00 Ba	−0.82 ± 0.00 Ba	−0.82 ± 0.00 Ba
NoFert	No sanitizer	−0.26 ± 0.44 Aa**	−0.04 ± 0.58 Aa	−0.47 ± 0.36 ACac	−0.82 ± 0.02 BCa
	PAA	−0.51 ± 0.31 Aa**	−0.82 ± 0.00 ACb	−0.66 ± 0.26 ACbc	−0.82 ± 0.00 Ba
	Cl	−0.55 ± 0.34 Aa**	−0.77 ± 0.05 Bb	−0.82 ± 0.00 Bb	−0.82 ± 0.00 Ba

^*a*^ Each separated based on their sanitizer condition: peracetic acid (PAA) or chlorine (Cl) at 20 ppm each or no sanitizer, which served as control. Limit of detection (LOD): −0.78 log CFU/tube. *N* = 9 per treatment combination. Uppercase letters represent significant differences between days in drip tubes within each treatment (fertilizer/sanitizer) combination. Lowercase letters represent significant differences across treatment combinations for the same day and fertilizer. Asterisks indicate significant differences among the fertilizer groups on day 0.

**Fig 2:**
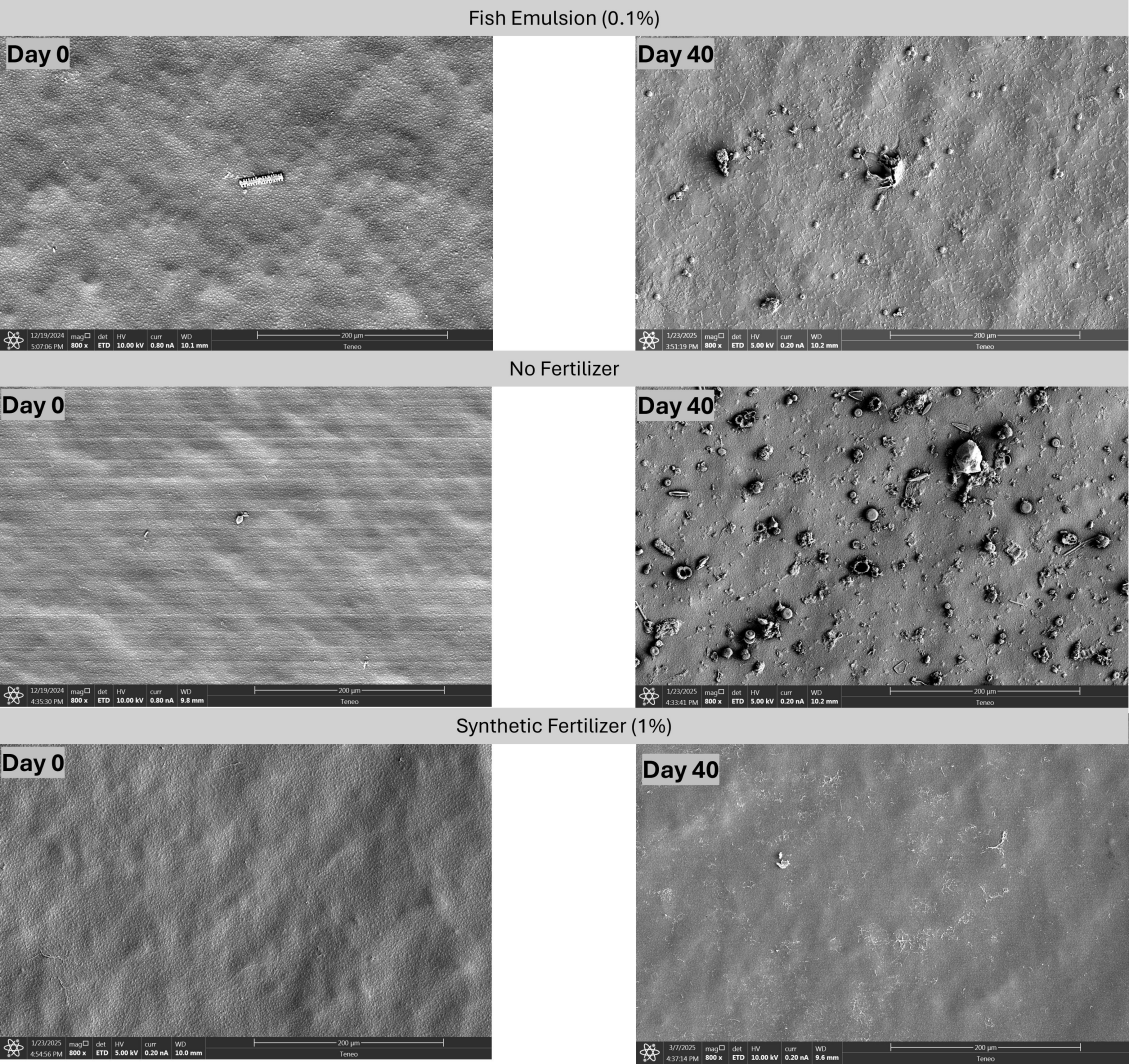
Scanning electron microscopy images of drip tubes (1.27 cm internal diameter – 0.25 cm^2^) treated with pond water + 0.1% (vol/vol) fish emulsion, pond water with no fertilizer, and pond water + 1% (vol/vol) synthetic fertilizer days 0 and 40 without sanitizer treatment. Pictures taken at magnification of ×800. Scale bar: 200 µm.

**Fig 3:**
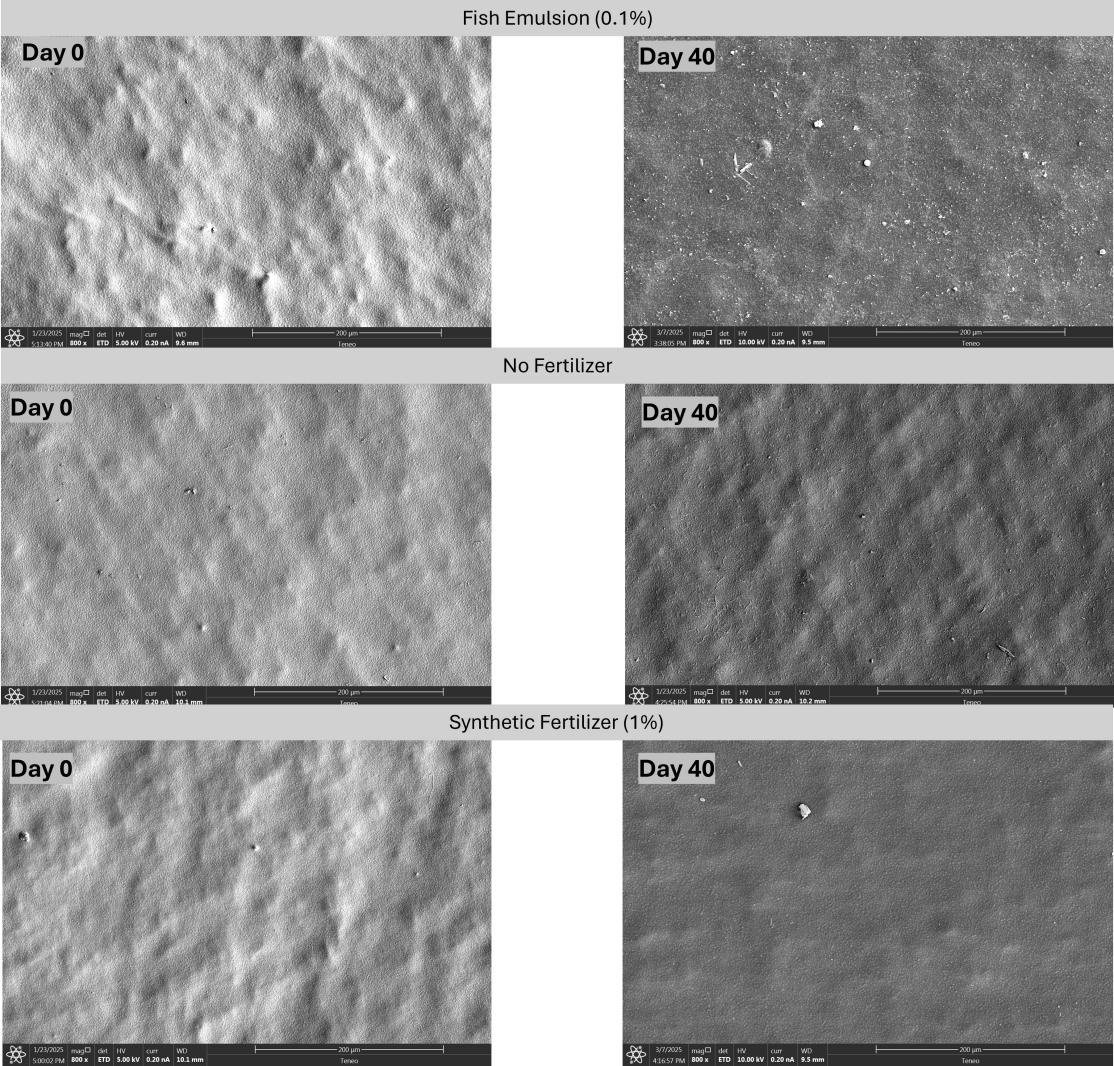
Scanning electron microscopy images of drip tubes (1.27 cm internal diameter – 0.25 cm^2^) treated with pond water + 0.1% (vol/vol) fish emulsion, pond water with no fertilizer, and pond water + 1% (vol/vol) synthetic fertilizer days 0 and 40 with 20 parts per million of peracetic acid as a sanitizer treatment. Pictures taken at magnification of ×800. Scale bar: 200 µm.

**Fig 4:**
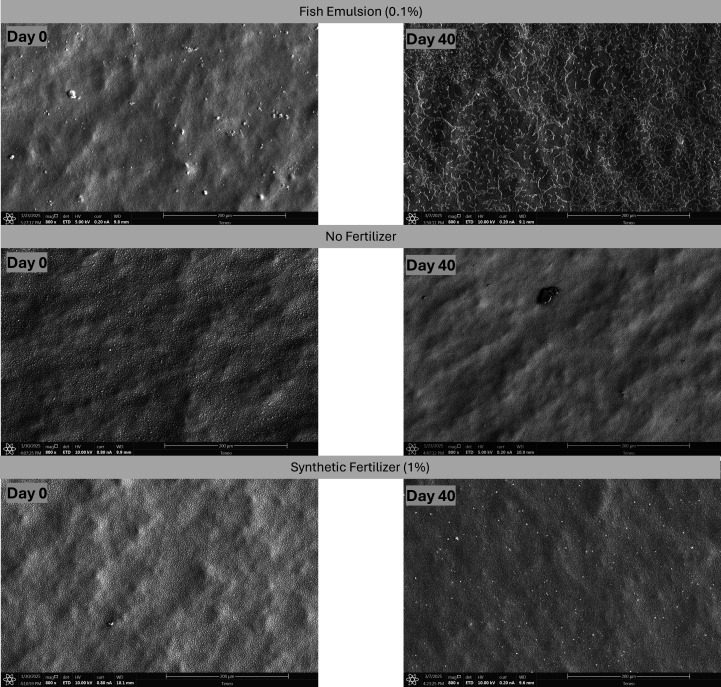
Scanning electron microscopy images of drip tubes (1.27 cm internal diameter – 0.25 cm^2^) treated with pond water + 0.1% (vol/vol) fish emulsion, pond water with no fertilizer, and pond water + 1% (vol/vol) synthetic fertilizer days 0 and 40 with 20 parts per million of chlorine as a sanitizer treatment. Pictures taken at magnification of ×800. Scale bar: 200 µm.

Populations in the tubing significantly increased in the O treatments without sanitizers (*P* ≤ 0.001) on day 7, reaching 5.45 log CFU/tube. They remained constant on day 14 (5.49 log CFU/tube), then gradually decreased on day 40 to 4.29 log CFU/tube (Table 2). Biofilm formation was evident based on the scanning electron microscopy (SEM) images, which illustrate the aggregation of cells and films formed on the drip tubes (Fig. 2; Fig. S1). In the O–PAA treatment combination, populations significantly increased (*P* = 0.002) to 1.49 log CFU/tube on day 7. It remained constant at 1.51 log CFU/tube, then decreased to 0.40 log CFU/tube on day 40 (Table 2). The start of a biofilm formation was evident in the PAA-treated samples with O fertilizers. However, no clear EPS structure was observed (Fig. 3; Fig. S2). Similarly, in the O–Cl treatment combination, *Salmonella* levels increased to 0.43 log CFU/tube then gradually decreased to 0.09 and −0.75 log CFU/tube on days 14 and 40, respectively (Table 2). Figure 4 and Fig. S3 illustrate a substantial aggregation of cells.

The NoFert treatment with no sanitizer-treated pond water (PW) showed a dynamic change in *Salmonella* levels on the tubing over the experimental period. Initially, *Salmonella* levels slightly increased to −0.04 log CFU/tube on day 7 (*P* = 0.89), followed by a gradual decrease to −0.47 log CFU/tube by day 14, and eventually reaching levels below the limit of detection (LOD) on day 40 (Table 2). Freshwater microorganisms such as diatoms are illustrated in the SEM images for the NoFert—no sanitizer treatment combinations throughout the days with no clear bacterial attachment represented (Fig. 2). In the NoFert–PAA treatment group, *Salmonella* populations significantly decreased (*P* = 0.001), falling below the LOD at day 7. Approximately 33% (3/9) of the NoFert–PAA tubing samples still showed at least one CFU of *Salmonella* using either enumeration methods; however, by day 40, *Salmonella* was undetectable in all samples (Table 2). SEM analysis revealed that diatoms and freshwater microorganisms adhered to the tubing throughout the experiment, along with dispersed rod-shaped cells; however, a mature *Salmonella* biofilm development appeared limited or absent throughout (Fig. 3). For the NoFert–Cl treatment combination, *Salmonella* levels were below the LOD for all sampling days. SEM analysis showed diatoms and free cells attached to the tubing, but no clear EPS and subsequent biofilm formation was shown (Fig. 4). In the S-treated samples with and without any sanitizer, *Salmonella* levels on the tubing remained below the LOD throughout the experimental days (Table 2). No clear biofilm formation or attachment of other microorganisms was observed on the SEM images (Fig. 2 to 4).

### *Salmonella* biofilms in drip lines contaminated later irrigation events when no sanitizers were injected

When the drip tubes were subsequently exposed to a new batch of PW on day 3 to simulate another irrigation event, it resulted in the cross-contamination for the water samples in the O (4.52 log CFU/mL) and the NoFert (0.97 log CFU/mL) treatments. Although it did not result in the contamination of water with tubing injected with S (levels below LOD; Table 3). *Salmonella* populations in the water for the O with no sanitizer treatment were significantly higher on day 3 (4.52 log CFU/mL; *P* = 0.0003) than population levels on day 0 (2.32 log CFU/mL). Bacterial concentrations decreased to 3.73 log CFU/mL and 3.86 log CFU/mL on days 7 and 10, respectively. Populations continued to decrease, reaching 3.4 log CFU/mL on day 17 in the PW circulated in the O with no sanitizer treated tubing with or without fertilizer throughout the experimental days (Fig. 1, Table 3). *Salmonella* remained detectable in the water samples for the NoFert treatments with no sanitizers on day 3 (0.97 log CFU/mL) and day 7 (−0.40 log CFU/mL) but then decreased to levels below the LOD for the remainder of the sampling days. *Salmonella* populations significantly decreased (*P* ≤ 0.0001) on day 3 to levels below the LOD and populations remained undetectable throughout the experimental procedure for the S treatments with no sanitizer (Table 3).

**TABLE 3:** *Salmonella* population (log CFU/mL) mean ± standard deviation in water samples over time at different fertilizer conditions: 0.1% vol/vol fish 2-4-1 emulsion (O), 1% v/v synthetic liquid 4-0-8 fertilizer (S), or pond water only (NoFert)^*a*^

Fertilizer	Sanitizer	Day 0	Day 3	Day 7	Day 10	Day 14	Day 17
O	No sanitizer	2.32 ± 0.08 Aa*	4.53 ± 1.43 Bca	3.76 ± 0.36 ABCa	3.86 ± 1.13 BCa	3.39 ± 0.08 ABCa	3.44 ± 1.07 ABCa
	PAA	2.31 ± 0.05 Aa*	−1.04 ± 0.02 Bb	1.54 ± 0.65 Ab	−1.05 ± 0.00 Bb	1.15 ± 0.83 Ab	−1.05 ± 0.00 Bb
	Cl	2.34 ± 0.05 Aa*	−0.97 ± 0.21 BCDb	−0.27 ± 0.78 ADc	−1.05 ± 0.00 BDb	−0.65 ± 0.44 ACc	−1.05 ± 0.00 BDb
S	No sanitizer	2.06 ± 0.22 Aa*	−1.05 ± 0.00 Ba	−1.05 ± 0.00 Ba	−1.05 ± 0.00 Ba	−1.05 ± 0.00 Ba	−1.05 ± 0.00 Ba
	PAA	2.13 ± 0.17 Aa*	−1.05 ± 0.00 Ba	−1.05 ± 0.00 Ba	−1.05 ± 0.00 Ba	−1.05 ± 0.00 Ba	−1.05 ± 0.00 Ba
	Cl	2.09 ±0.32 Aa*	−1.05 ± 0.00 Ba	−1.05 ± 0.00 Ba	−1.05 ± 0.00 Ba	−1.05 ± 0.00 Ba	−1.05 ± 0.00 Ba
NoFert	No sanitizer	2.33 ± 0.12 Aa*	0.97 ± 0.24 Aa	−0.40 ± 0.47 Bb	−0.85 ± 0.23 Ba	−1.04 ± 0.02 Ba	−1.05 ± 0.00 Ba
	PAA	2.12 ± 0.73 Aa*	−1.05 ± 0.00 Bb	−1.05 ± 0.00 Bb	−1.05 ± 0.00 Bb	−1.05 ± 0.00 Bb	−1.05 ± 0.00 Bb
	Cl	2.34 ± 0.10 Aa*	−1.05 ± 0.00 Bb	−1.05 ± 0.00 Bb	−1.05 ± 0.00 Bb	−1.05 ± 0.00 Bb	−1.05 ± 0.00 Bb

^*a*^ Each separated based on their sanitizer condition: peracetic acid (PAA) or chlorine (Cl) at 20 ppm each or no sanitizer, which served as control. Limit of detection (LOD): −1.00 log CFU/mL. *N* = 9 per treatment combination.Uppercase letters represent significant differences between days in drip tubes within each treatment (fertilizer/sanitizer) combination. Lowercase letters represent significant differences across treatment combinations for the same day and fertilizer. Asterisks indicate significant differences among the fertilizer groups on day 0.

### PAA and Cl at 20 ppm effectively prevented cross-contamination of *Salmonella* from the tubing to the subsequent irrigation water but did not completely eliminate the biofilms already formed in the tubing

On day 3, both PAA and Cl effectively reduced *Salmonella* populations to levels below the LOD for all fertilizer treatments in water samples (Table 3). The observed effect was consistent across days 10 and 17. However, on days 7 and 14, the O and PAA treatments resulted in detectable counts of 1.54 log CFU/mL and 1.15 log CFU/mL, in water samples, respectively. The O and Cl treatment combination exhibited similar behavior with detectable levels on days 7 and 14 at −0.26 and −0.65 log CFU/mL, respectively (Table 3). The S and NoFert treatments with PAA and Cl resulted in levels below the LOD on days 7 and 14.

## DISCUSSION

The increase in recovery of bacterial cells from the drip tubes on day 0 for the O compared with the S and NoFert may be attributed to the non-uniform bacterial attachment on the tubing and the ingredients found in the fish emulsion, including hydrolyzed fish. Numerous studies have shown that soil microbial diversity is significantly enhanced when treated with organic fertilizers, as opposed to conventional mineral fertilizers (33–36). For example, the meta-analysis conducted by Bebber and Richards (36) reported data from 37 research articles and revealed that organic fertilizers significantly enhance both functional and taxonomic diversity of soil microbes compared with unfertilized control soils and mineral fertilizers. Others have reported the role of strains, surface type, temperature, and other ecological features in the formation of biofilms on abiotic surfaces (14, 37–39). For example, Contreras-Soto et al. (37) reported that *Salmonella* biofilms exposed to river water exhibited significant structural variations compared with those formed in laboratory conditions. The structural variations observed through SEM indicated that environmental factors in river water, such as organic matter and nutrients, contribute to the non-uniform formation of biofilms on surfaces. Additionally, the fish emulsion treatment, in this study, promoted greater bacterial attachment to the tubing after one hour of circulation compared with the synthetic 4-0-8 fertilizer or surface water alone. This enhanced attachment may be attributed to the higher carbon content in the fish emulsion, which could have initiated the early, reversible stages of biofilm formation (39–41). The sustained increase in *Salmonella* populations on the tubing in the O and no-sanitizer treatments over time suggests that continuous water circulation within the drip lines, regardless of fertilizer presence, supported *Salmonella* survival and attachment. The slight decline in *Salmonella* populations observed on days 7 (3.73 log CFU/mL) and 10 (3.86 log CFU/mL) compared with the peak on day 3 (4.52 log CFU/mL; *P* = 0.0003), in the O, no sanitizer treatment, suggests that although the bacteria remained viable in the system, part of the reduction may be attributed to water circulation causing detachment of cells from biofilms or dilution within the irrigation loop. Moreover, the minimal decline in *Salmonella* levels despite nearly 20 days without water input indicates robust biofilm formation on the tubing surface. This is further supported by the SEM images, which illustrate a more mature biofilm formed on the tubes (Fig. 2; Fig. S1).

The decrease in *Salmonella* populations within the tubing observed in the NoFert–no sanitizer treatment combination indicates that, with the absence of water circulation (from day 17-40), *Salmonella* biofilms were unable to sustain themselves by the end of the experimental period. This observation aligns with findings from previous studies, which have demonstrated that biofilm formation by *Salmonella* on abiotic surfaces can be highly variable and influenced by multiple conditions. The production of EPS is crucial for biofilm persistence, but various factors such as nutrient availability, microbial competition, and environmental factors such as temperature and relative humidity can significantly impact biofilm stability (42–44). Figure 2 further supports this concept by illustrating the presence of other diatoms and non-rod-shaped materials on the tubing. This suggests that the microbial community within the tubing was diverse and included various freshwater organisms. The presence of these organisms implies potential competition for resources, which could inhibit *Salmonella* biofilm formation. Studies have shown that microbial interactions, including competition and cooperation, play a critical role in shaping biofilm structure and stability (45, 46). For example, Behringer et al. (45) highlighted the strong conservation of bacterial communities associated with diatoms, suggesting that these interactions can influence biofilm formation and persistence. Hence, the inability of *Salmonella* to form sustained biofilms in the NoFert treatment can be attributed to the complex interplay of environmental factors and microbial competition. Understanding these interactions is essential for developing effective strategies to control biofilm formation in natural and engineered systems.

In this study, *Salmonella* populations dropped below the LOD in both tubing and water samples when no sanitizer was applied, in the presence of the 4-0-8 synthetic fertilizer (Tables 1 and 2). This clearly indicates that synthetic fertilizers inhibit *Salmonella* attachment and subsequent growth in the system. SEM images showed that no organisms, besides algae-like filaments from the PW, have attached to the tubing within the S treatments over time (Fig. 2). Many crop production researchers have reported that long-term N fertilization significantly alters the microbial environment, leading to shifts in microbial community composition and diversity. These changes disrupt the natural balance of microbial communities, affecting nutrient cycling and microbial interactions (47, 48). Studies have reported that the gradual release of N in the form of ammonia through synthetic fertilization can suppress the growth and survival of *Salmonella* species (49–51). The N content in the 4-0-8 synthetic fertilizer used in this study consists of 4.18% nitrate-N and 0.32% ammonium-N (Table S1). When ammonium-N dissolves in water, it undergoes a chemical transformation that can produce nitrite (NO_2_), nitric oxide (NO), and other gaseous forms of N (52–55) that can have antimicrobial effects against *Salmonella enterica* (56–59). Additionally, microbial dynamics can be influenced when synthetic salts from fertilizers are added to the irrigation systems. It can either induce osmotic stress, prompting bacteria to form biofilms as a protective mechanism (60), or inhibit the survival of certain pathogens, such as *Salmonella* (61). Water electroconductivity (EC) measures the ionic strength of water; hence, as the amount of synthetic salt increases, EC increases (62). The addition of 1% (vol/vol) 4-0-8 synthetic fertilizer in this study greatly elevated the EC of the water at various times (Table S2). To add, many have reported the irreversible correlation of bacterial concentrations and EC (63–65). For example, McEgan et al. (63) reported the highly significant inverse correlation (*P* = 0.0001) with *E. coli* concentrations as EC level increases. Osmotic conditions may have been altered due to the elevated EC levels caused by the addition of the 4-0-8 fertilizer used in this study, potentially increasing microbial stress, thereby affecting their viability. In practical irrigation systems, EC is not static. It typically increases following fertilizer application due to the influx of dissolved salts and subsequently decreases as the system is flushed. This study aimed to simulate continuous flushing to understand its impact on EC dynamics and pathogen dynamics. However, water pressure may also play a critical role in this process, potentially influencing the effectiveness of flushing and the distribution of synthetic fertilizers. These proposed mechanisms, including the role of nutrient availability in fish emulsion and potential antimicrobial effects of synthetic fertilizers, remain hypotheses and should be further evaluated through targeted experiments. The insights, however, underscore the need to consider both flushing regimes and hydraulic conditions when evaluating the behavior of foodborne pathogens in fertigation systems, particularly throughout seasonal re-use of lines. Finally, the material type of the irrigation line may have impacted the attachment and subsequent biofilm formation of *Salmonella*. For example, Ivers et al. (14) reported that *Salmonella* attachment and biofilm characteristics were significantly impacted by surface type and incubation time, where the highest biofilm formation was observed on nylon surfaces across all time points (2, 24, and 96 h), whereas the least biofilm formation was observed on HDPE surfaces.

Bacterial concentrations remained constant in water circulated in the O with no sanitizer-treated tubing with or without fertilizer throughout the experiment (Table 2). This indicates that the continuous water circulation in the line supported the stable survival and attachment of *Salmonella*. The consistency in bacterial concentrations indicates that *Salmonella* have formed biofilms on the tubing surfaces, corroborating the previously discussed SEM tubing data for the O treatment. Biofilms are protective layers that bacteria create, making them more resistant to environmental stress and treatments. Based on Fig. S1 presenting *Salmonella* biofilm formation with O treatment on day 40, a visible crack is present in the tubing within the biofilm, which was not observed on day 14. This suggests that *Salmonella* survived over time in the tubing, even in periods of non-use. This finding is particularly relevant in agricultural settings, where irrigation lines are often reused across multiple growing seasons in which they may remain inactive in-between crop production stages. The reintroduction of water and nutrients could promote new biofilm formation (39, 66–68) and potentially reintroduce *Salmonella* contamination in subsequent seasons. The switch between the O fertilizer (days 7 and 14) and no fertilizer injection in the water (days 3, 10, and 17) suggests that the biofilm could thrive regardless of nutrient availability from the constant addition of fertilizers.

*Salmonella* remained detectable in the water samples for the NoFert treatments with no sanitizers on day 3 and day 7 but then decreased to levels below the LOD for the remainder of the sampling days, indicating that the conditions in the NoFert-treated tubing became less favorable over time. Nutrient depletion, accumulation of waste products, or changes in environmental conditions can negatively impact the microorganisms’ survival, all of which could occur on the field. Figures 2 to 4 illustrate the potential for cellular attachment within the tubing line when no fertilizer is injected, and only a low abundance of cells is observed, with minimal adhesion evident in the cell structure, compared with when pond water is used in combination with injected fish emulsion. In agricultural or environmental contexts, this suggests that the absence of fertilizers (NoFert) may initially support microbial growth, but over time, the lack of nutrients can lead to planktonic cells reproducing but becoming incapable of sustaining themselves and then dying off. Once the cells attached, they may not be able to form a mature biofilm. However, based on evidence from this study, the line can still harbor *Salmonella* over 14 days.

On days 3, 10, and 17, both PAA and Cl effectively reduced *Salmonella* populations to levels below the LOD for all fertilizer treatments in water samples (Table 2). This indicates that water treated with 20 ppm of either PAA or Cl can limit cross-contamination of *Salmonella* from tubes to water. However, on days 7 and 14, the O-PAA and O-Cl treatments resulted in detectable counts in water samples compared to the NoFert and S fertilizer treatments with either PAA or Cl. Biofilm formation was evident in the PAA-treated samples with O fertilizers in Fig. 3 and Fig. S2. This indicates that although PAA may have limited cross-contamination from the drip tubes treated with fish emulsion to the circulated water treated with the sanitizer, it did not eliminate the bacteria attached to the tubing. Consequently, cross-contamination from the dispersed biofilms occurred in the water circulated with the fertilizer that was not treated. PAA is known for its strong oxidizing properties, which can disrupt cell walls and cellular components of microorganisms (27, 30). However, biofilms provide a protective barrier that can limit the penetration and effectiveness of PAA (13, 42). This barrier, composed of EPS including proteins and polysaccharides, can prevent PAA from reaching and eliminating all bacteria within the biofilm, leading to persistent contamination. The protective nature of biofilms allows bacteria to survive and potentially detach, leading to cross-contamination in untreated areas. PAA is also biodegradable, rapidly decomposing into acetic acid, hydrogen peroxide, and water (30). The rapid biodegradability of PAA means that it breaks down quickly, reducing its effective concentration over time (69). As PAA decomposes, the residual acetic acid and hydrogen peroxide may not be sufficient to completely eradicate biofilms, as noticed by the results of this experiment. The chemical stability of biofilms, combined with the rapid degradation of PAA, means that biofilms can persist within the irrigation loop system in this study, even after PAA treatment. The O and Cl treatment combination exhibited similar behavior, albeit to a lesser extent. This indicates that Cl was more effective in inhibiting *Salmonella* growth, potentially due to its strong oxidizing properties and ability to generate reactive oxygen species (ROS) such as hypochlorous acid, superoxide, and hydroxyl radicals (28). These ROS induce oxidative stress, damaging proteins, lipids, and DNA within *Salmonella* cells, leading to their effective elimination. Nonetheless, Fig. 4 and Fig. S3 illustrate a more substantial cell aggregation compared with Fig. 3 and Fig. S2, which shows non-uniform biofilm formation. This suggests that Cl was more effective in inhibiting *Salmonella* growth, but not biofilm formation along with other microorganisms potentially present in freshwater when fish fertilizers are present in-line. Stress responses to PAA and Cl further complicate the eradication of biofilms as microorganisms can adapt to these sanitizers, enhancing their resilience (70). Within a biofilm, bacteria can either up- or down-regulate genes, produce extracellular matrix components, and activate antibiotic resistance mechanisms such as efflux pumps, even without antibiotic exposure, while also expressing elevated levels of virulence factors (71–73). These adaptations make biofilm-associated cells more resilient than their planktonic counterparts (74, 75). In the context of the food-to-fork chain, this mobility increases the risk of introducing resilient pathogens to previously uncontaminated surfaces or products. Their ability to survive harsh conditions and evade standard sanitization measures makes them particularly dangerous during this phase, amplifying the potential for widespread contamination and infection (70). This highlights the need for additional or combined treatments to effectively manage biofilm formation within irrigation systems. Additionally, in field settings, drip tubing, such as that used in southern Georgia, often remains unflushed and in place until the next growing season, which may span 20–40 days depending on management practices, crop type, and time of year. According to industry practices, tubing is either replaced each season or retained until it no longer functions effectively. Hence, the potential for cross-contamination observed in the O and NoFert treatments is particularly important, as residual water and nutrients left in tubing over extended periods could facilitate microbial survival, biofilm development, and subsequent contamination of irrigation water in the following season.

Our results presented the effectiveness of PAA and Cl in limiting biofilm cross-contamination within irrigation lines in a closed-loop system, simulating 40 days of irrigation production. However, several limitations should be noted. The experiment was conducted on a small scale in a laboratory setting, which does not fully replicate field conditions, particularly the high-water volumes and pressures typically used in agricultural operations. Therefore, field-based studies are necessary to evaluate the performance of these sanitizers under real-world conditions. Field systems typically operate at flow rates of 300–600 gal/min, whereas our model employed ~5 gal/min. This difference in hydraulic conditions can strongly influence biofilm development and sanitizer efficacy (76–78). It should also be noted, however, that irrigation distribution systems closest to the crop many times are in this configuration, as water flow is distributed throughout the entire acreage being irrigated. Therefore, the findings presented here should be interpreted as a first step toward understanding pathogen behavior in irrigation water systems. Because *Salmonella* is a BSL-2 pathogen, establishing a leak-free system at field-equivalent flow rates presents significant biosafety challenges. For this reason, the current model was designed to provide a controlled, reproducible environment that allows initial investigation of *Salmonella* persistence and biofilm formation under safe laboratory conditions. Future work will focus on increasing flow rates and employing non-pathogenic surrogates, which will allow testing under conditions that more closely resemble field systems while maintaining biosafety. The initial inoculation level in this study was 2 log CFU/mL, which is higher than levels typically encountered in real-world agricultural settings; however, such levels were used in the lab study to enumerate and observe sanitizers' effects on microbial load over time and to ensure bacterial attachment on the surface of the tubing. In this study, we tested only one concentration of PAA and Cl (20 ppm) to establish proof of concept for sanitizer performance within the irrigation loop model. This level was selected because 20 ppm is the concentration most commonly applied in commercial practice and recommended by manufacturers (27, 79–81). Future in-field studies will be expanded to include a range of concentrations and contact times in order to more fully characterize sanitizer performance under real-world operating conditions. Environmental factors such as elevated field temperatures, which were not replicated in this room-temperature study, may also impact biofilm formation and should be explored in future research. Finally, no attempt was made to quantify or semi-quantify biofilm formation; the SEM images were included solely to provide a visual representation of biofilm presence on the surfaces examined. These qualitative observations complement the sequencing-based findings by illustrating the physical manifestation of microbial colonization, reinforcing the relevance of biofilm formation in shaping surface microbiomes despite the lack of quantitative assessment. Nonetheless, this study underscores the importance of flushing techniques as part of chemigation management strategies to mitigate microbial buildup within irrigation systems. Other sanitation interventions such as chemical shock flushes, hot air blowouts, and other hurdle approach line-cleaning methods can disrupt biofilm development (82–84). For example, the application of physical methods for biofilm removal, including ultrasound, electric fields, plasma, magnetic fields, and irradiation (85). Ultrasonic sterilization, when combined with chemical treatments, has proven to be a safe and effective technique for degrading biofilms and enhancing the disruption of biofilms formed by pathogens such as *Staphylococcus aureus* and *Salmonella* spp. (86). These empirical findings underscore that thoughtfully applied chemical flushing regimens are essential for controlling microbial persistence, especially in systems prone to biofilm-related risks.

### Conclusion

Our findings suggest that fish emulsion fertilization enhances biofilm growth in a loop setting over time. Although PAA and Cl decrease *Salmonella* population and cross-contamination during irrigation, they are less effective afterward when biofilm is formed. Cl has a longer-lasting bactericidal effect than PAA in drip tubes treated with fish fertilization. PAA and Cl are effective at reducing population and biofilm formation when no fertilizer is added to the water. Regardless of sanitizer treatment, the 1% of 4-0-8 synthetic fertilizer treatment limited *Salmonella* growth and survival of other freshwater organisms in the line. Biofilm formation is not continuously formed on the tubing, and the presence of diatoms may provide locations for bacterial aggregation and biofouling. The incomplete elimination of bacteria in the NoFert tubing resulted in cross-contamination in the water circulated with no sanitizer. The residual bacteria in the biofilm can detach and contaminate the circulated water, especially when untreated fertilizers are introduced. Overall, these findings underscore the need for effective sanitation practices in irrigation systems to prevent the biofilm formation and spread of pathogenic bacteria such as *Salmonella*. Understanding these dynamics can help in developing better strategies for managing microbial contamination in agricultural settings.

## MATERIALS AND METHODS

### Bacterial culture

For this study, a four-serotype cocktail of *Salmonella enterica* was used: *S. enterica* Enteritidis (2020AM-1539-2020 Peach outbreak), *S. enterica* Poona (ATCC BAA-3139-2010 Cucumber outbreak), *S. enterica* Newport (2020AM-0919-2020 Onion outbreak), and *S. enterica* Montevideo (ATCC BAA-710-1993 Tomato outbreak). Strains were adapted to 80 parts per million (ppm) rifampicin and stored at −80°C in glycerol stocks. Before inoculation, 10 µL of each strain was transferred individually and grown in tryptic soy broth with rifampicin (TSBR; Difco, Becton Dickinson Co., Sparks, MD, USA) for 24 h at 37°C three times consecutively. To create a bacterial lawn, after the third transfer, 250 µL of each strain was inoculated onto tryptic soy agar plates with 80 ppm of the rifampicin (TSAR; Difco, Becton Dickinson Co) then incubated at 37°C for 24 h. Bacterial cells were harvested by flooding each plate with 10 mL buffered peptone water (BPW; Difco, Becton Dickinson Co.) and dislodging cells with a cell spreader. Equal volumes (3 mL) of each serotype were combined to create the four-serotype cocktail which was used for inoculation. To determine the initial *Salmonella* populations, the combined cocktail was serially diluted in 0.1% (wt/vol) peptone water (Difco, Becton Dickinson Co), plated on TSAR, and incubated at 37°C for 24 h prior to enumeration. The initial *Salmonella* cocktail population determined was at 10 log CFU/mL.

### Irrigation water collection and inoculation

Surface water was collected using a peristaltic pump (GEOPUMP2, Geotech Environmental Equipment, Inc., Denver, CO, USA) from a pond that is used for irrigating crops in southern Georgia Tri County area over the summer of 2024 and used for all challenge studies. Once collected, water jugs were placed on ice and transported to the lab to be frozen at −20°C until usage. Water was stored at −20°C until it was thawed at 4°C for immediate usage. Pond water (PW) was either injected with 1% (vol/vol) synthetic liquid 4N-0P-8K fertilizer (S; R.W. Griffin, Ty Ty, GA, USA; Table S2) or with 0.1% (vol/vol) fish emulsion (O; 2N-4P-1K; Ocean Crest Seafoods Inc., Gloucester, MA, USA). PW with no fertilizers (NoFert) was used as a control. Each type of water was inoculated with a 2 log CFU/mL cocktail of rifampicin-adapted *Salmonella* cultures mentioned previously by diluting the cocktail in 9 mL of PW to reach a final concentration of ~ 2 log CFU/mL in the sample water.

### Drip line preparation in a loop system

Each set of polyethylene drip tubes (1.27 cm internal diameter; NDS Inc., Lindsay, CA, USA) with no perforations was connected to a 110V magnetic pump which had a water flow of 19 L/min (5 gal/min) (YaeKoo, Amazon, Seattle, WA, USA) and a pre-assembled one-gallon jug with a ½ inch spigot kit. These components were placed in a large black 26 × 18 × 12," heavy-duty tote to control the water flow and prevent any leakage since pathogens were used for this study. The drip tubes were aseptically cut to approximately 8.5 inches (22 cm) each and then connected using in-line couplers (1/2" Drip Irrigation Coupling Fitting, Amazon). Each treatment combination included one magnetic pump connected to a pre-cut tube and a water jug drilled and attached to a ½ inch spigot with a barbed valve. To connect the tube set with the magnetic pump, a ½ inch female fitting was attached to each side of the pump using ½ inch female threaded PVC fittings, ensuring secure attachment. Then, a ½ inch drip line coupling was connected to the female PVC thread on each side, ensuring tight connections. On the upper side, the drip tube leading to the entire set of tubing was connected, and on the lower side, a ~3 inch drip tube was connected to the barbed valve from the jug (Fig. S4). This in-lab irrigation loop system enabled us to evaluate irrigation events using water inoculated with biosafety level II pathogens

### Irrigation water circulation in-line

On day 0, each jug was filled with 1.5 L of inoculated PW, tailored to its respective fertilizer condition. The spigot was then turned on to initiate water circulation for 1 h, simulating a continuous irrigation event. This setup ensured that surface water was the first and only source of contamination on that day. To simulate post-irrigation water conditions, the inoculated water was removed from the jugs. Non-inoculated PW from the same source was then continuously replaced according to specific schedules: with its respective sanitizer condition on days 3, 10, and 17, or with its respective fertilizer condition on days 7 and 14. This process began after the initial inoculation on day 0, mimicking a typical crop production month on the farm (Fig. 1).

### Sanitizer treatments

For each treatment combination set, the water was either treated with 20 ppm of PAA (Sanidate 12.0, BioSafe Systems, East Hartford, CT, USA), 20 ppm of CL (65% Granular Cal Hypo, Aqua Org, Amazon), or left as untreated surface pond water to serve as a control. Sanitizer concentrations were determined based on common practices and manufacturer’s recommendations and were assessed by using the FAS-DPD Chlorine/Bromine test kit (LaMotte, Chestertown, MD, USA) and PAATest Kit (Thermo Fisher Scientific, Waltham, MA, USA) for free Cl and PAA, respectively, before each circulation day. The water was circulated in-line for 5 minutes, followed by a 1 h period of stagnant contact time (Fig. 1).

### Processing and microbial determination

Populations in water were determined on days 0, 3, 7, 10, and 17, whereas populations in tubes were determined on days 0, 7, 14, and 40 per the following: 20 mL of water were collected from each jug onto 50 mL conical tube. The latter was repeated three times to indicate three technical replicates for each treatment/day combination. On day 0, water samples were collected at time 0 and after 1 h of circulation, then processed for microbial testing. Although the remainder of the days, water samples were collected after the irrigation stopped (after 1 h). For the days when the circulated water was treated with sanitizers, 0.2 mL of neutralizer was added to the conical tubes. The neutralizer was prepared by dissolving 28g of 97% granular sodium metabisulfite (Fisher Scientific, Pittsburgh, PA, USA) in 1 L of deionized water. Samples were then mixed and diluted in 0.1% (wt/vol) peptone water (Difco, Becton Dickinson Co) as needed and spiral plated (EDDY JET2, v1.0, IUL Instruments, Barcelona, Spain) in duplicate on xylose lysine tergitol 4+rifampicin (XLT4R; Difco, Becton Dickinson Co.). Populations were determined after incubation at 37°C for 24–48 h. Simultaneously, 10 mL of each sample was filtered using 0.45 µm membrane filters (MilliporeSigma, Burlington, MA, USA) and plated on XLT4R for 24–48 h. Limit of detection (LOD) of water samples: −1 log CFU/mL.

The remaining populations attached to the drip tubes were determined by randomly selecting three tubing from each treatment combination and aseptically cutting it to four equal parts. Each cut part was washed with 25 mL of sterilized deionized water to remove any planktonic cells. Biofilms were dislodged using HiCap swabs (BLU-10HC, World Bioproducts, Woodinville, WA, USA). The swab solution was later diluted in 0.1% (wt/vol) peptone water, as needed, and spiral plated in duplicate on XLT4R at 37°C for 24–48 h. The remainder of the swab solution (6 mL) was filtered using 0.45 µm membrane filters (MilliporeSigma) and plated on XLT4R at 37°C for 24–48h (LOD of tube samples: −0.78 log CFU/tube).

### Scanning electron microscopy

Undisturbed tubing was imaged each sampling day by aseptically cutting square sections (0.25 cm^2^) and analyzed by SEM imaging to determine the biofilm structure throughout the production process. To fix the biofilms on the surface, 200 µL of 10% formalin (wt/vol) (Fisher Scientific) was added to the cut tubing for 10 min. After 10 min, the tubing was washed with 500 µL of sterilized deionized water. Samples were kept at 4°C until examination. Fixed tubes were then sputter-coated with gold at the following settings WD 12.5 mm, 60 s, 15 mA (SPI sputter coater, Structure Probe, Inc., West Chester, PA, USA) and examined with a scanning electron microscope acceleration voltage of 500 V–30 kV, at working distance 10 mm (FE-SEM Thermo Fisher Teneo, Waltham, MA, USA).

### Data analysis

A completely randomized design was used with three samples per each three biological replicates analyzed (*N* = 27) for each treatment combination. To capture low levels of inoculated or attached *Salmonella* in the water or tubes, respectively, both enumeration by plating and membrane filtration methods were used simultaneously. Plate counts were deemed acceptable following guidelines for spiral plates use according to the US Food and Drug Administration’s Chapter 3 of the Bacteriological Analytical Manual (87) and standard guidelines for filter enumeration (88). Based on the Shapiro-Wilk test, the distribution of the *Salmonella* populations across the different treatments was not normally distributed; therefore, a Kruskal-Wallis test followed by a Steel-Dwass *post-hoc* analysis was used in R v4.3.3 (89) to compare differences in means between treatments and between each treatment across days. A Wilcoxon rank-sum test was used when comparing between two independent groups. *P*-values below 0.05 were considered significant. When *Salmonella* was not detected by plating or membrane filtration, a value of −1.05 log CFU/mL or −0.78 log CFU/tube was assigned to each water or tube sample, respectively, for data analysis. The negative values represent samples in which bacterial counts either fell below the limit of detection or were extremely low (1 cell detected between the three technical replicates). In biological terms, a negative log value does not indicate a negative number of bacteria; rather, it results from assigning a small, fixed value to allow logarithmic transformation for statistical analysis. For example, a value of −0.78 log CFU/tube corresponds to approximately 0.17 CFU/tube, whereas −0.31 log CFU/tube corresponds to roughly 0.49 CFU/tube.

## SUPPLEMENTAL MATERIAL

Supplemental material aem.01974-25-s0001.docx Tables S1 and S2; Fig. S1 to S4.

ASM does not own the copyrights to Supplemental Material that may be linked to, or accessed through, an article. The authors have granted ASM a non-exclusive, world-wide license to publish the Supplemental Material files. Please contact the corresponding author directly for reuse.
