# Supplementary material for: Efficacy of peracetic acid and chlorine in managing Salmonella biofilms in irrigation loop systems
Source: Appl Environ Microbiol. 2025 Dec 19;92(1):e01974-25. doi: 10.1128/aem.01974-25 (PMC12838213; doi:10.1128/aem.01974-25)
Supplement: Supplemental material — Tables S1 and S2; Fig. S1 to S4. [file aem.01974-25-s0001.docx]

**Supplementary Materials**

| **Table S1:** Synthetic liquid fertilizer component analysis used for this study. Sample analyzed by Water Agricultural Labs Inc. 257 Newton Rd, Camilla, GA 31730-1653 | |
| --- | --- |
| **Analyte** | **Result (%)** |
| Nitrogen - Total | 4.50 |
| Nitrate - Nitrogen | 4.18 |
| Ammonium - Nitrogen | 0.32 |
| Urea - Nitrogen | 0.00 |
| P_2_O_5_ - Total | 0.00 |
| K_2_O - Total | 9.38 |

| **Table S2:** Conductivity (µs/cm) of pond water only, pond water with 1% (v/v) synthetic 4-0-8 liquid fertilizer, or pond water with 0.1% (v/v) fish 2-4-1 emulsion after 1 min or 1 h of injection. Conductivity and pH were measured using HQ4300 Portable Multi-Meter (Hach, Loveland CO, USA). | | |
| --- | --- | --- |
| **Sample** | **Contact time with water** | **Conductivity (µs/cm)** |
| Pond Water - Only | 1 min | 241 |
| Pond Water + 1% of 4-0-8 | 1 min | 5180 |
| Pond Water +0.1% of 2-4-1 | 1 min | 287 |
| Pond Water + 1% of 4-0-8 | 1 h | 5040 |
| Pond Water + 0.1% of 2-4-1 | 1h | 243 |

**Fig. S1:** SEM image of the inside surface of a drip tube (1.27 cm internal diameter – 0.25 cm^2^) treated with pond water + 0.1% fish emulsion on days 0, 7, 14, and 40 **without sanitizer treatment.** Pictures taken at magnification of 2500x. Scale bar: 50 µm. Biofilm formation and growth is evident over time.


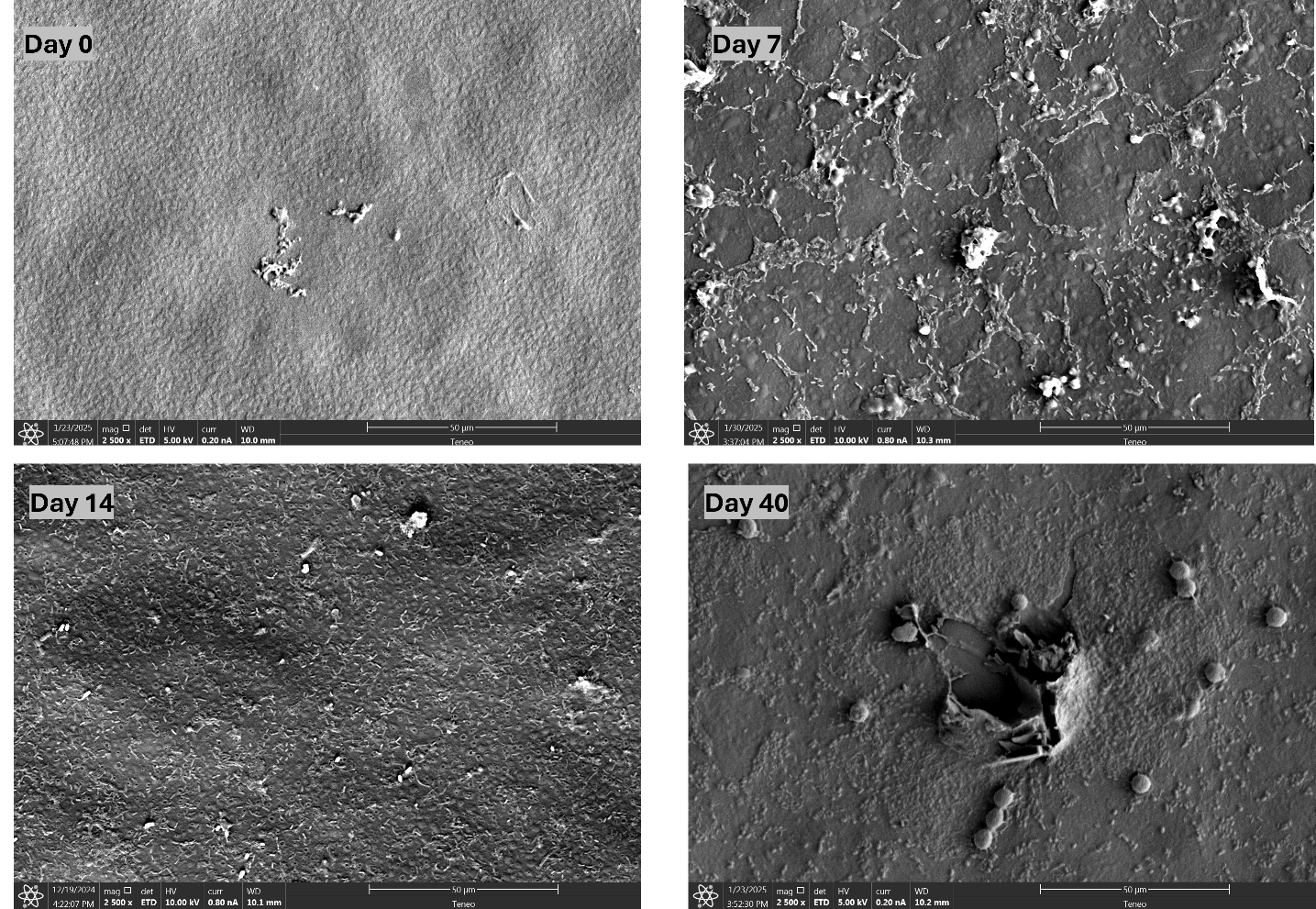


**
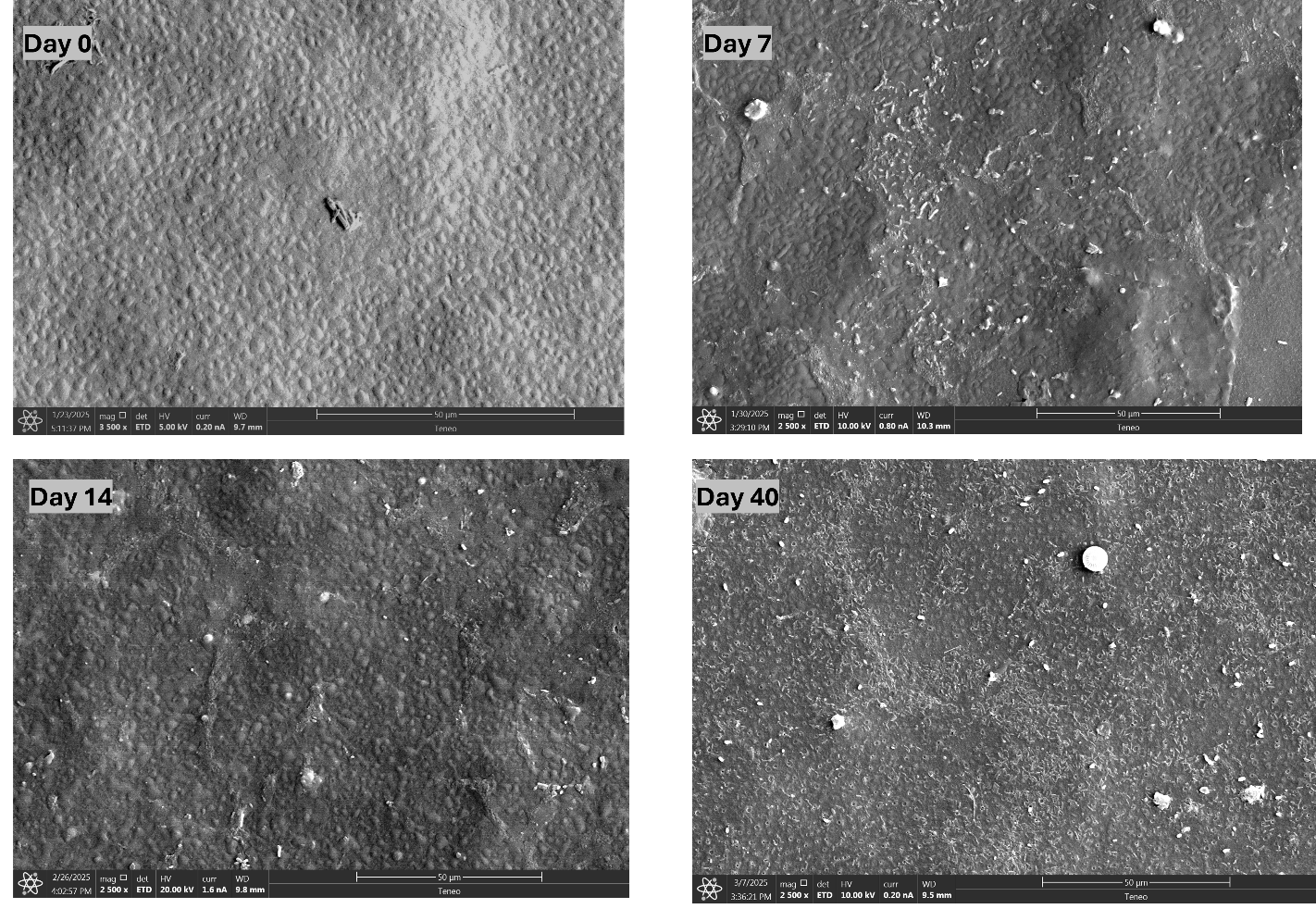
Fig. S2:** SEM image of the inside surface of a drip tube (1.27 cm internal diameter – 0.25 cm^2^)) treated with pond water + 0.1% fish emulsion on days 0, 7, 14, and 40 with **peracetic acid** treatment. Pictures taken at magnification of 2500x. Scale bar: 50 µm.

**Fig. S3:** SEM image of the inside surface of a drip tube (1.27 cm internal diameter – 0.25 cm^2^) treated with pond water + 0.1% fish emulsion on days 0, 7, 14, and 40 with **chlorine** treatment. Pictures taken at magnification of 3500x. Scale bar: 50 µm

**
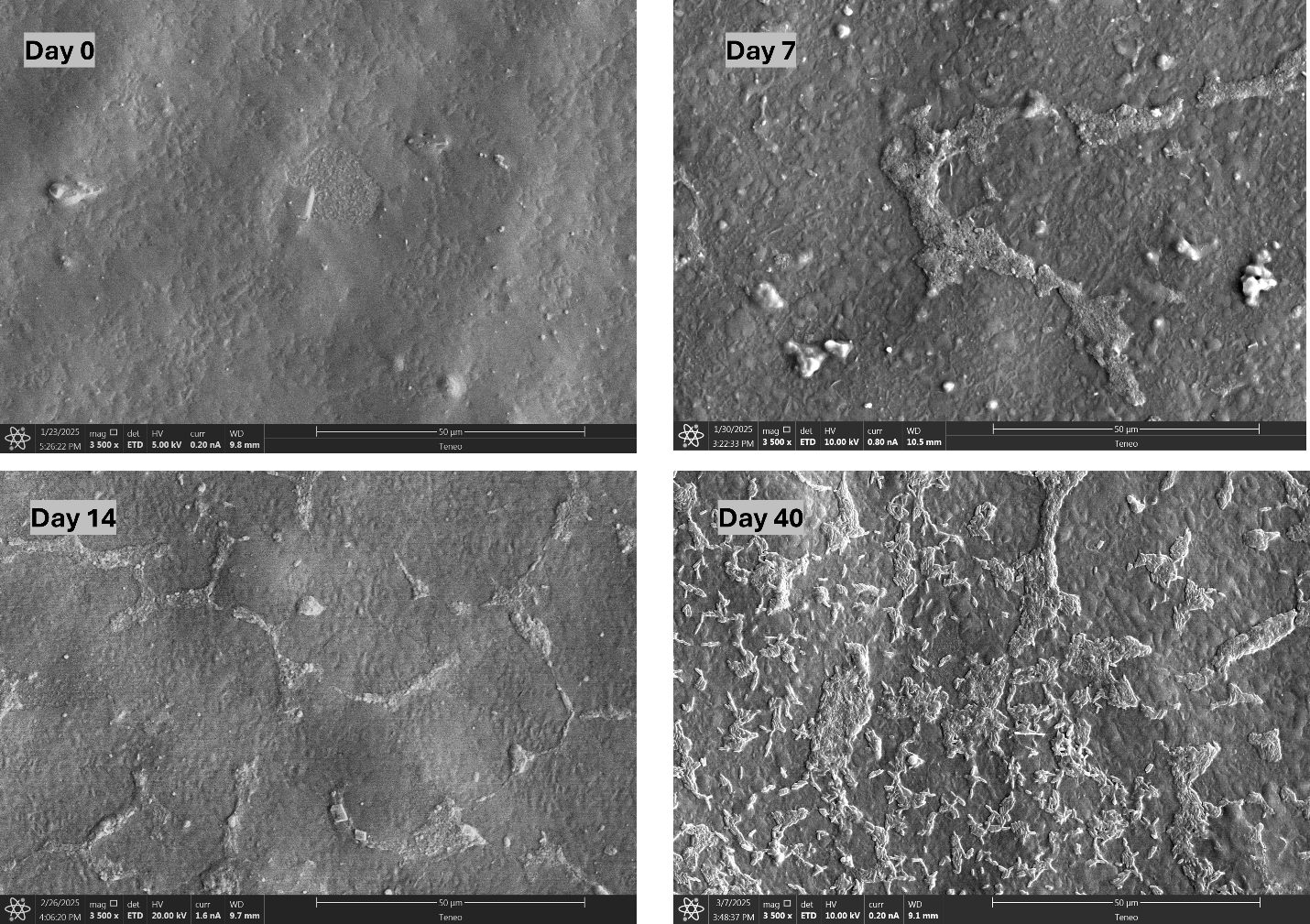
**


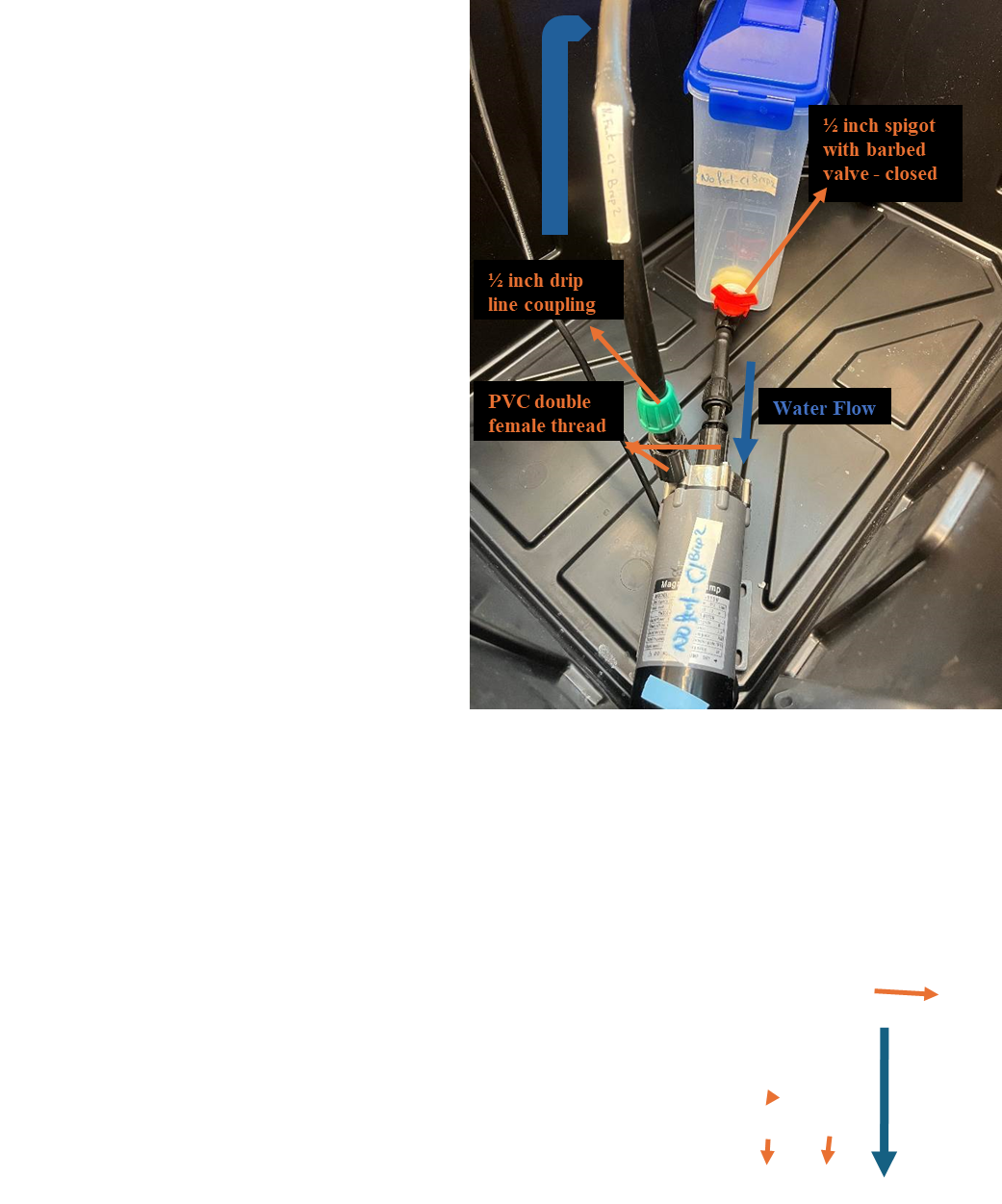
**Fig. S4:** Example Setup of polyethylene drip tubing (without perforations) and pump used for water circulation in a single treatment combination. PVC= polyvinyl chloride
